# A smart and sustainable pathway for abatement of single and binary mixtures of dyes through magnetically retrievable Ca_4_Fe_9_O_17_ anchored on Biochar matrix

**DOI:** 10.1038/s41598-023-40077-w

**Published:** 2023-08-09

**Authors:** Gaurav Yadav, Soumya Ranjan Mishra, Vishal Gadore, Nidhi Yadav, Md. Ahmaruzzaman

**Affiliations:** Department of Chemistry, National Institute of Technology Silchar, Silchar, Assam 788010 India

**Keywords:** Pollution remediation, Nanoscale materials

## Abstract

In this work, the author developed Ca_4_Fe_9_O_17_/biochar (CFB) via a green method through a facile co-precipitation procedure involving egg shells as calcium precursor and investigating its performance in single as well as binary solution of methylene blue (MB) and rhodamine B (RhB). The CFB nanocomposite was characterized by XRD, SEM, TEM, XPS, Raman, FTIR, BET, and VSM. ESR studies show the presence of hydroxyl (·OH) and superoxide (O_2_·¯) radicals, which are primary radical species for pollutant degradation. The average crystalline size of CFB nanocomposites was found to be 32.992 nm using XRD, whereas TEM analysis indicates a particle diameter of 35–36 nm. The degradation efficacy of MB and RhB dyes was achieved at 99.2% and 98.6%, respectively, in a single solution, whereas 99.4% and 99.2%, respectively, in a binary solution within 36 min. Additionally, an iron cluster was formed during the degradation process of MB dye. The degradation of organic contaminants and generation of iron clusters from the degraded dye products were both expedited by the remarkable extension effect of the Ca_4_Fe_9_O_17_ in the CFB nanocomposites. The three processes were achieved using CFB nanocomposite: (1) the advanced oxidation process; (2) degradation of MB and RhB dye in single as well as binary solution with enhanced efficiency, (3) the production of the iron cluster from degraded products. Thus, these three steps constitute a smart and sustainable way that leads to an effective effluent water treatment system and the generation of iron clusters preventing secondary pollution.

## Introduction

There are limited resources for clean water, but due to anthropogenic activity, these resources are contaminated, making the situation worse^1,2^. In recent years, the abatement of dyes has garnered attention because of their massive production, carcinogenic nature, and slow biodegradation. Dye concentration in water endangers water safety for aquatic animals as well as human beings. Dye disposal in nearby water sources causes numerous problems, including increased chemical oxygen demand, toxicity, unpleasant smell, and color. The principal techniques for the removal of wastewater consists of bioremediation^3^, selective membrane process^4^, adsorption^5^, photocatalysis^6^, and advanced oxidation process^7^.

Over recent years, advanced oxidation processes (AOP) have emerged as cutting-edge and effective techniques for abatement of contaminants in wastewater. AOP helps in oxidizing dyes and breaks them into simple and low molecular weight substances with lower toxicity, such as carboxylic acid, aldehydes, and inorganic compounds^8^. The AOP process generally needs an activator like hydrogen peroxide^9^ and persulfate^10^, which helps in the generation of free radicals, which further beneficial for removal of organic contaminants. The OH͘ radical generation can be enhanced by H_2_O_2_, O_2_, O_3_, and UV radiation. Recently persulfate, peroxymonosulfate (PMS), and H_2_O_2_ have gained attention in the field of AOP for the degradation of pollutants. Although PMS and persulfate have some limitations due to the presence of sulfur, which leads to the generation of secondary pollutants in large amounts. Therefore, H_2_O_2_ is commonly used as an oxidant species in the advanced oxidation process because the H_2_O_2_ dissociation leads to the formation of oxygen and water^11^. Because of its non-toxicity, H_2_O_2_ is considered a green oxidizing agent. H_2_O_2_ itself does not reduce the organic contaminations. Currently, numerous materials have illustrated the H_2_O_2_ activation properties. Nevertheless, their poor catalytic efficiencies have hampered their future usage as H_2_O_2_ activators.

A number of studies show that biochar-based composite materials are efficiently used for the degradation of contaminants from water^12^. Biochar (BC) is a carbon-rich substance that is prepared by the pyrolysis of biomass under a specific temperature without oxygen^13^. The carbon-rich material (BC) shows promising activity to adsorb inorganic and organic pollutants from wastewater. Moreover, biochar has unique surface characteristics, such as increased porosity, huge surface area, and functional group abundance, which all promote the rapid abatement of pollutants^14^. Biochar supports other metal oxide catalysts because of abundant functional groups, chemical conductivity, and stability^15^. Moreover, biochar slows the recombination of the electron-hole pairs and traps impurities on its surface, increasing the catalyst’s effectiveness^14,16^.

Nanotechnology has developed rapidly in recent years due to small particle sizes (1–100 nm)^17^. Nanotechnology is widely applicable in numerous areas, including organic transformation, environmental remediation, sensing, etc.^18,19^. Iron-based nanoparticles possess remarkable characteristics, including reactivity, biocompatibility, and thermal and chemical stability^20,21^. According to reports, the uniquely developed iron-based nanoparticles/BC composite might inherit the benefits of iron and biochar to improve catalytic performance. Combining biochar with iron-based materials to boost H_2_O_2_ activation efficacy is a growing trend because of the efficient H_2_O_2_ activation capability of iron-based catalysts^22^. Spinal ferrites, compared to other iron-based compounds, have gained focus due to their superior stability, strong magnetism, and reactive activity^23^. Oxygen molecules are incorporated on the tetrahedral sites with the cationic metals on the octahedral sites to construct the spinel ferrite type material MFe_2_O_4_ (M = Zn^2+^, Mg^2+^, Ni^2+^, Ca^2+^, Cu^2+^, Ba^2+^, etc.)^24^. CaFe_2_O_4_ is a typical spinel ferrite that comprises no toxic metal, has low-cost, high availability, is environment friendly, and has excellent optical properties^25^. Simultaneously CaFe_2_O_4_ has a few specific characteristic properties, such as being easily separable from the reaction medium, reusable for multiple reaction cycles, and many more. Therefore, CaFe_2_O_4_ is significantly used for water remediation. CaFe_2_O_4_’s orthorhombic crystal structure consists mostly of deformed FeO_6_ octahedral exchanging boundaries and 8-coordinated calcium molecules at vertices, and its unusual Ca–Fe–O framework allows it to be used in a variety of processes^26^. However, CaFe_2_O_4_ displays a low activity due to high charge recombination and charge transfer, which limits its applications. A special p-type character in CaFe_2_O_4_ is thought to be responsible for invalid hole formation on the surface of the CaFe_2_O_4_, which is responsible for poor quantum yield^27^. Similar to CaFe_2_O_4_, there is a new class of ferrites material like Ca_4_Fe_9_O_17_, have recently been investigated for photocatalysis^28^. This class possesses the same characteristics as CaFe_2_O_4_ due to Ca–Fe–O ternary system. Likewise, the Ca_4_Fe_9_O_17_ possesses high electron-hole recombination. The previous study shows that Ca_4_Fe_9_O_17_ possesses a crystalline phase and mesoporous surface^28^. Various researchers used this ferrite material for rechargeable batteries^29^, calculating thermodynamic properties^30^, and Gibb’s free energy^31^. However, there is a lack of research regarding this ferrite material, especially for degradation.

Organic pollutants that are present in water bodies are not easily degradable due to their resilience towards microorganisms. MB and RhB are the common cationic dyes that are discharged from various textile industries and contaminate water. These organic dyes cause serious environmental as well as biological concerns. MB is also a synthetic medicine that is used to treat methemoglobinemia, psychological problems, the neurological system, and even malaria^32^. It has a cascade of immediate negative consequences, including anemia at very high dosages. In humans, acute cationic dye exposure may result in narcosis, jaundice, heat stroke, etc.^33^. Because of their non-biodegradability, the dyes are toxic and carcinogenic for humans^34^. The interest in removing pollutants from wastewater continues to increase and is a never-ending source of fascination for environmentalists. In the previous literature, the researcher mainly focuses on single-dye degradation. But the real wastewater generated from textile industries contains many toxic dyes, challenging wastewater purification issues. Recently there has been an increasing trend of the degradation of binary dye solution. Different materials, such as BiVO_4_/CeO_2_^35^, BiOCl^36^, CuFe_2_O_4_^37^, and Ce-MoS_2_^38^, were used for the degradation of a mixture of dyes. But all these materials exhibit low catalytic efficiency toward dye mixture.

Millions of tons of eggshells are produced each year, contributing to global biowaste. The eggshell has a similar composition to the animal bone. However, the utilization of eggshells to develop green catalysts for environmental remediation has yet to be found. This work explains the successful synthesis of novel green Ca_4_Fe_9_O_17_/Biochar (CFB) by a facile co-precipitation method using eggshells as calcium precursors. Saw dust biochar was effectively used to increase catalysts' functional group and porous structure. A number of characterizations have been done to know the morphology, functional groups, catalyst properties, and degradation intermediate. To assess the potential of CFB nanocomposite for dye degradation, the effects of a number of important variable such as H_2_O_2_ concentration, catalyst loading, dye concentration, and contact time were thoroughly investigated. In addition to that, our study illuminated the processes of H_2_O_2_ activation while creating a potential, eco-friendly approach to dyes eradication. Iron cluster formation from the degraded dye products does not results in secondary pollution.

## Materials and methods

### Materials

FeCl_3_ anhydrous and NaOH were obtained from Sigma Aldrich. The eggshell was used as a calcium source. Saw dust was procured from a local furniture shop near NIT Silchar. Rhodamine B C_28_H_31_ClN_2_O_3_ C.I. 45170 and Methylene blue C_16_H_18_ClN_3_S.3H_2_O was obtained from SRL chemical, a certified reference material. All the chemicals were graded analytical. Distilled water obtained from Direct-Q (Millipore) was used in all experiments. Whatman paper of size 0.45µm was used to filter the solution mixture.

### Catalyst preparation

Egg shells and sawdust were washed multiple times to remove impurities and dried at 80 °C for 24–48 hours. After that, egg shells are ground into a fine powder and sieved using 250 µm. Simultaneously sawdust was also sieved using 250 µm. The fine powder of eggshell (CaCO_3_) is calcined at 800 °C for 4 hrs to get CaO. To generate Ca_4_Fe_9_O_17_/Biochar, the molar ratio of CaO powder and anhydrous FeCl_3_ was kept at 4:9, and the weight ratio was kept at 1:5, with one weight equivalent of biochar and 5 weight equivalents of combined mass of CaO and anhydrous FeCl_3_. Then CaO powder (2.80 g), anhydrous FeCl_3_ (13.48 g), and sawdust (3.25 g) were immersed in 50 mL of water and stirred for a half hour for effective mixing of chemicals. After stirring, the pH of the solution was adjusted by adding NaOH solution. After 4 h of heating at 80 °C, the solution was filtered and dried at 80 °C overnight in a hot air oven. The obtained material was pyrolyzed at 600 °C for 2 h to get the desired material (Ca_4_Fe_9_O_17_/Biochar). To investigate the comparison of biochar and Ca_4_Fe_9_O_17_/Biochar, pure sawdust was pyrolyzed at 600 °C for 2 h to obtain biochar named SDBC (sawdust biochar).

### Catalyst characterization

A Bruker 3000 Hyperion Microscope outfitted with a Vertex 80 FTIR spectrometer was utilized on the KBr pellets. The Phillips X’pert Pro MPD (multipurpose diffractometer) was used to perform powder XRD at a scan speed of 2°/min utilizing Cu K radiation (2 = 10–90). Jeol 6390LA/OXFORD XMX N is used for SEM-EDS analysis with accelerating voltage of 0.5 to 30 kV and magnification up to 30k. EDS has a resolution of 136 eV and an area detector of 30 mm^2^. HRTEM was investigated by Jeol/JEM 2100 (200 KV), consisting LaB6 electron gun having lattice resolution and point resolution is 0.14 nm and 0.23 nm, respectively. The free radical test has done by the ESR technique (JES-FA200). The VSM lakeshore model (7400 series) determines the magnetic properties of the catalyst. Nova Station B was used in the N_2_ atmosphere for N_2_ adsorption-desorption. Before that, the sample was degassed at 80 °C. XPS analysis was obtained from the Nexsa base model made by Thermo Fischer Scientific. FT-RAMAN spectrometer analysis is obtained using Bruker RFS with a wavelength of 50–5000 cm^–1^. HPLC with LCMS received from Agilent 6545XT AdvanceBio LC/Q-TOF to know the end products and understand the formation of the iron cluster. The dye concentration was measured by a Genesys 10S UV-Vis spectrophotometer.

### Catalyst activity

The stock solutions of 100 mL of different dyes were prepared for the experiment and kept in the dark. Before the AOP process, the solution was kept in the dark for adsorption-desorption equilibrium. No significant change is observed in dark conditions indicating that CFB nanocomposite does not show adsorption behavior. The experiment of the AOP was conducted in a beaker containing 20 ppm of dyes in a 50 mL solution with a particular amount of H_2_O_2_ and catalyst dosage. The reaction was kept at room temperature without pH adjustment. The dye intensity was measured using a UV-Visible spectrophotometer at 664 nm and 553 for MB and RhB dye, respectively. All the experiments were measured thrice, and the average values were considered. The efficiency of the dye degradation is calculated as Eq. (1).

1 $$\mathrm{Efficiency}\,\left(\mathrm{\%}\right)=\frac{{C}_{0}-C}{{C}_{0}}\times 100.$$

Here C_0_ is the initial absorbance of the pollutants, and C is the final absorbance of the catalyst.

The kinetics of the advanced oxidation process was measured using Eqs. (2) and (3).

2 $$\mathrm{ln}\frac{{C}_{0}}{{C}_{t}} =\mathrm{ kT},$$

3 $$\frac{1}{{C}_{t}}-\frac{1}{{C}_{0}}=kT.$$

Equation (2) represents the pseudo-first-order kinetics, whereas Eq. (3) denotes the pseudo-second-order kinetics of the degradation. C_0_ is the initial concentration at time (t)=0, and C_t_ is the final concentration at time t. k is the rate constant of the pseudo-first-order reaction.

## Results and discussion

### XRD

**Figure 1:**
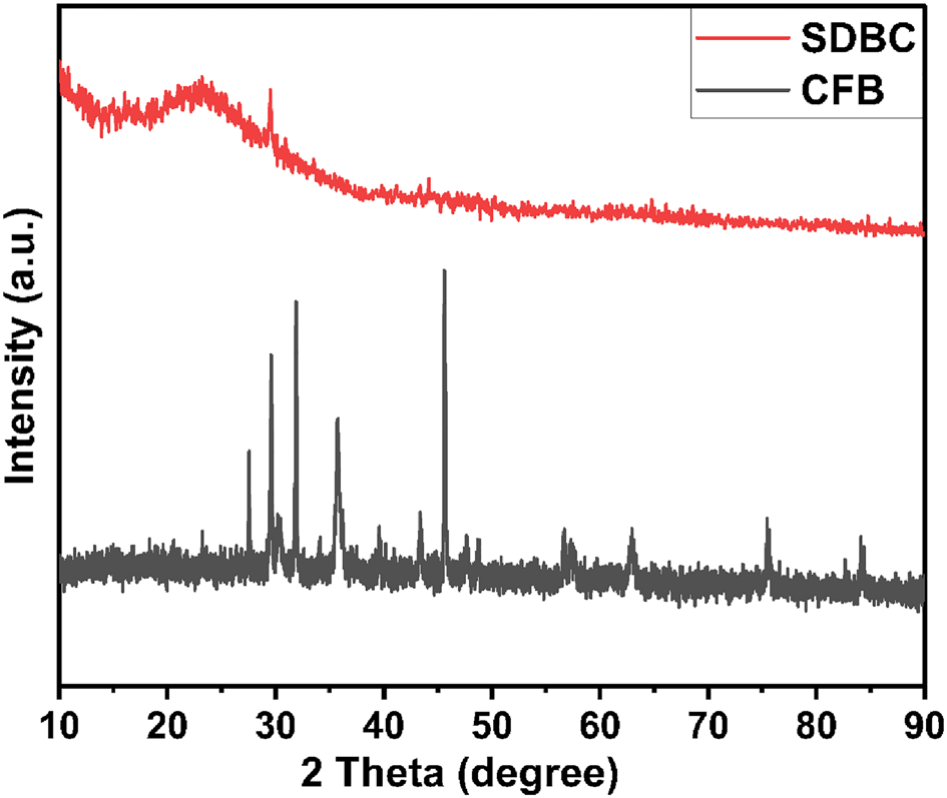
XRD pattern of CFB nanocomposite and SDBC.

The XRD pattern of SDBC and Ca_4_Fe_9_O_17_/Biochar (CFB) nanocomposite was shown in Fig. 1. In the XRD pattern of SDBC, a broad diffraction peak between 18 to 27° is observed, which is considered to be the amorphous structure of biochar^39^. Its amorphous nature was verified by the XRD pattern, which was characteristically broad, with the prominent peaks being Ca_4_Fe_9_O_17_^40^. The XRD data show the prominent peak of Ca_4_Fe_9_O_17_ at 31.91°, which matches with the JCPDS card no. 75-2421. The peaks on the CFB at 16.11°, 30.3°, 31.91°, 32.3°, 35.87°, 43.53°, and 45.69° corresponded to the Ca_4_Fe_9_O_17_ planes (002), (310), (004), (311), (313), (404), and (403), demonstrating the existence of monoclinic Ca_4_Fe_9_O_17_ [Space group C2{5}]. The slight change in the intensity of Ca_4_Fe_9_O_17_ indicates the successful insertion into biochar to form the CFB matrix^41^. The cell parameters of the corresponding Ca_4_Fe_9_O_17_ are a=10.44 Å, b=6.025 Å, and c=11.384 Å. The spinal phase of CaFe_2_O_4_ could not be found in this composite material.

Furthermore, The average crystallite size was calculated using the Debye-Scherrer equation^42^ from the full-width at half-maximum (FWHM) corresponding to the sharpest 2ϴ location at 31.91°. Debye- Scherrer’s formula (Eq. 4) may also be used to determine the size of a crystal;

4 $$\mathrm{D}=\frac{k\lambda }{\beta cos\Theta }.$$

Here, β is the full width at half maxima (FWHM), k is the shape factor, ϴ is Bragg’s angle, and λ is the wavelength. The average crystalline size of Ca_4_Fe_9_O_17_, as obtained by the Debye Scherer equation, was found to be 32.992 nm.

### SEM and TEM analysis

**Figure 2:**
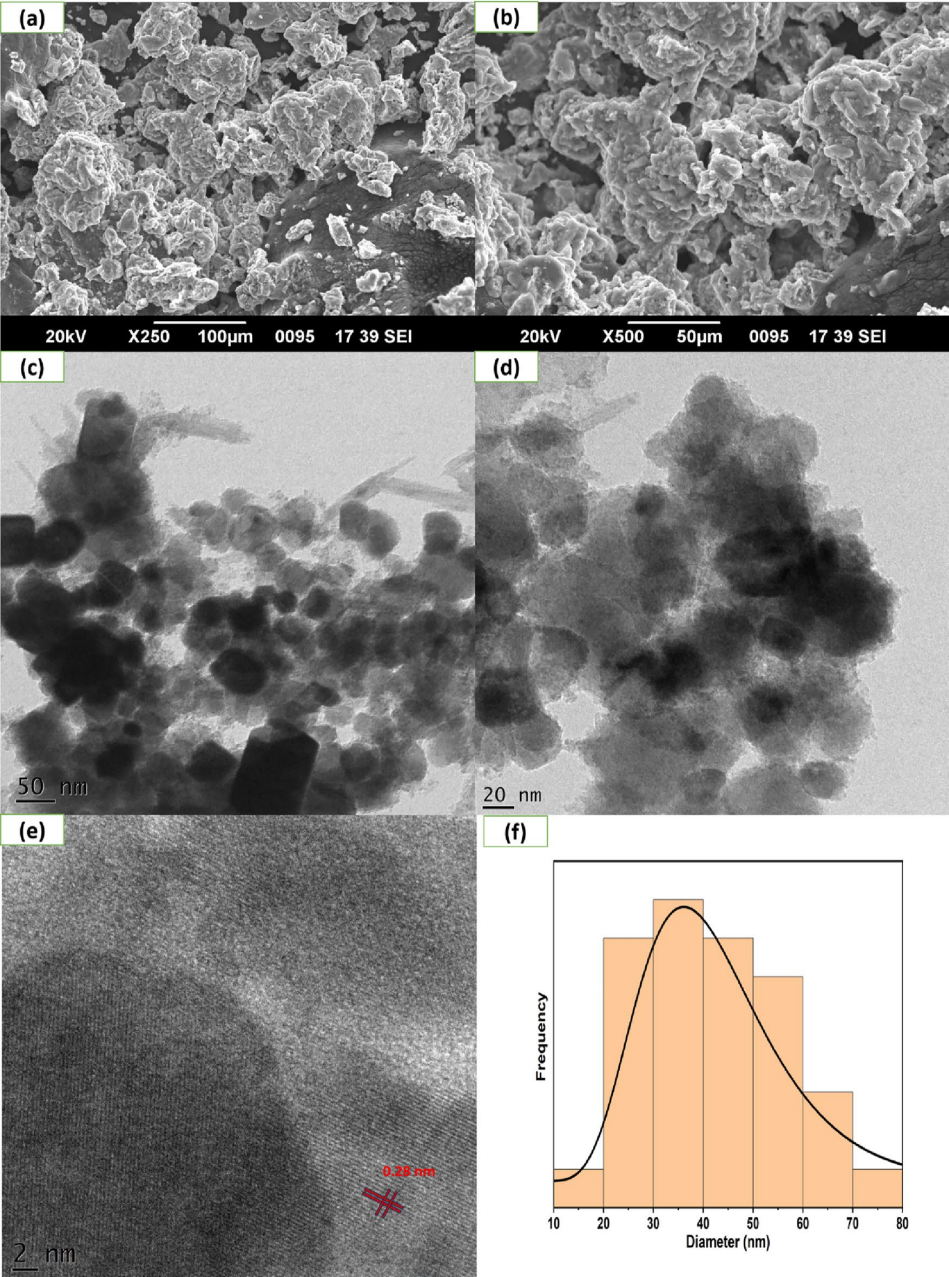
Demonstration of (**a**,**b**) SEM images, (**c**,**d**) TEM, (**e**) HRTEM, (**f**) and particle size distribution curve of CFB nanocomposite.

Scanning electron microscopy (SEM) was performed to investigate the morphology of the CFB nanocomposite (Fig. 2a,b). SEM images show the rough surface of the synthesized nanocomposite, which may be due to particle agglomeration, as reported in the previous literature^43^. A large number of gaseous by-products, such as hydrogen, carbon dioxide, and monoxide, are released during synthesis, which accounts for the porous structure. Consequently, the degradation efficiency of the CFB nanocomposite is enhanced.

Furthermore, TEM was employed to study the morphology of the nanocomposite and to learn more about its structure (Fig. 2c,d). TEM images show that Ca_4_Fe_9_O_17_ nanoparticles were found to be homogeneously distributed on the biochar matrix. Nanoparticles were aggregated to form a cluster with a spherical shape, rod shape, and a relatively even distribution. The particle size distribution curve shows that the average particles are sized between 35 and 36 nm (Fig. 2f). This data was consistent with the average particle size of the Ca_4_Fe_9_O_17_ obtained by the XRD spectra. The high-resolution TEM (HRTEM) picture revealed a 0.28 nm lattice fringe (Fig. 2e) assigned to the (004) plane of Ca_4_Fe_9_O_17_ (JCPDS No. 75–2421).

### BET analysis

**Figure 3:**
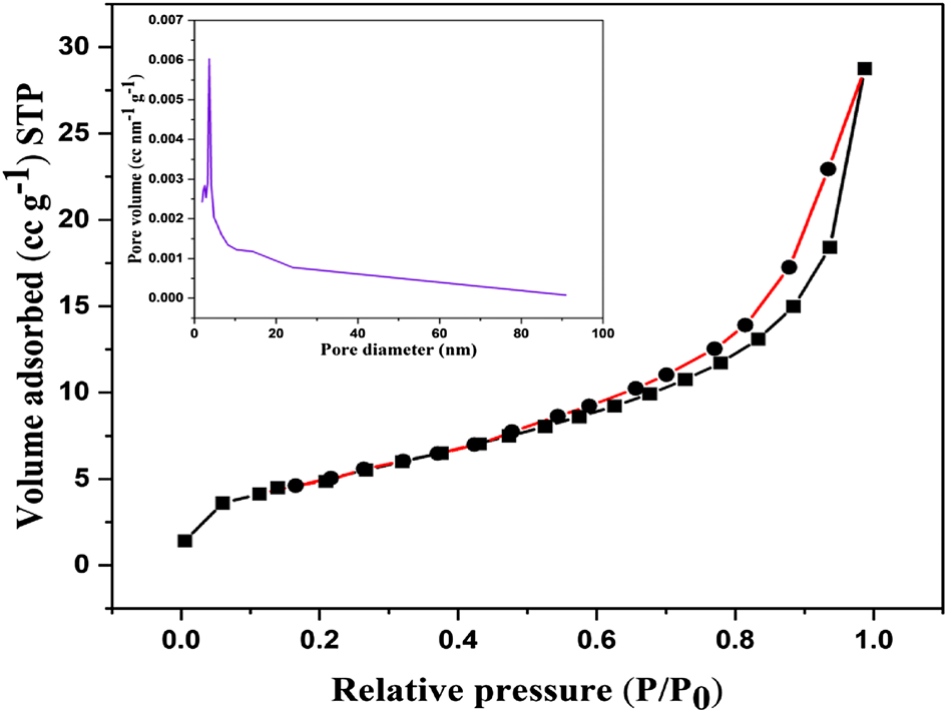
Pore size distribution curve with the adsorption-desorption curve of CFB nanocomposite.

In this work, BET analysis was done to evaluate the surface area and porosity of the CFB nanocomposite. The pore size distribution and surface area of the nanocomposite were calculated by N_2_ adsorption data. Fig. 3 displays the mesoporous surface of the CFB nanocomposite and shows type IV with an H3 hysteresis loop. The surface area was 21.380 m^2^/g for the CFB nanocomposite, whereas pore volume and pore diameter were 0.046 cc/g and 3.646 nm, respectively. The inclusion of Ca_4_Fe_9_O_17_ during the carbonization process led to a decrease in the matrix shrinkage of the biochar, which resulted in a greater specific surface area for the CFB nanocomposite^44^. As the specific surface area increases, the activity of the nanocomposite increases because of the availability of surface-active sites. It was demonstrated by the BET analysis that dye molecules were promptly incorporated into the mesoporous surface of the CFB nanocomposites. As a result, CFB nanocomposites’ surface area and pore size distribution data demonstrated improved results for the removal of dyes.

### VSM analysis

**Figure 4:**
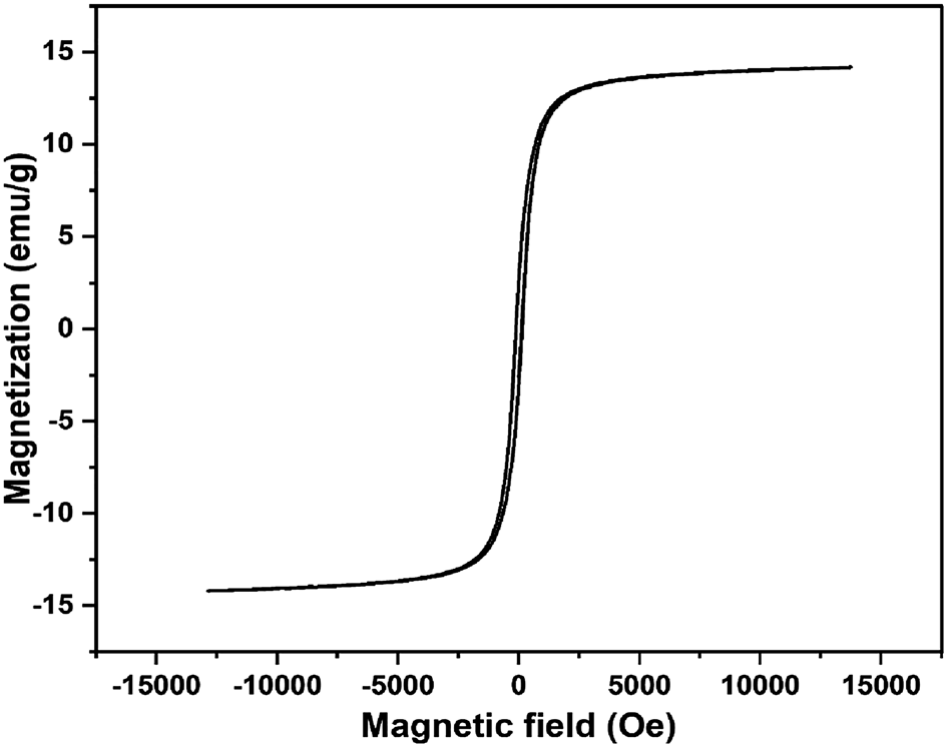
The hysteresis loop of CFB nanocomposite showing strong attraction in the magnetic field.

In order to analyze the magnetic properties of the CFB nanocomposite, VSM analysis was conducted. At room temperature, field measurements versus initial magnetization were done using a magnetometer that swept the field from − 13,000 to 13,000 Oe (Fig. 4). The superparamagnetic nature of the CFB nanocomposite was confirmed by the S-shaped hysteresis loop^45^. Additionally, CFB nanocomposite was readily isolated from an aqueous medium using a magnet due to its high saturation magnetization of 14.20 emu g^–1^ coercivity 133.14 Oe and retentivity 2.92 emu g^–1^. From the VSM studies, it was clear that the nanocomposite shows superparamagnetic behavior. Because of dipolar interactions, the demagnetizing effect diminishes the coercivity and magnetic squareness (M_R_/M_S_) values of connecting superparamagnetic atoms, bringing them closer to the value of 0.5 for non-interacting superparamagnetic atoms^46^. The obtained results were consistent with a magnetic squareness value of 0.21, which indicates that 90% of magnetism was dissipated upon magnetic field removal^47^. Since the obtained squareness value was 0.21, it may be inferred that superparamagnetism was exhibited by the CFB nanocomposite. The degree of magnetocrystalline anisotropy of a particle is proportional to its superparamagnetic properties^48^.

### XPS spectra

**Figure 5:**
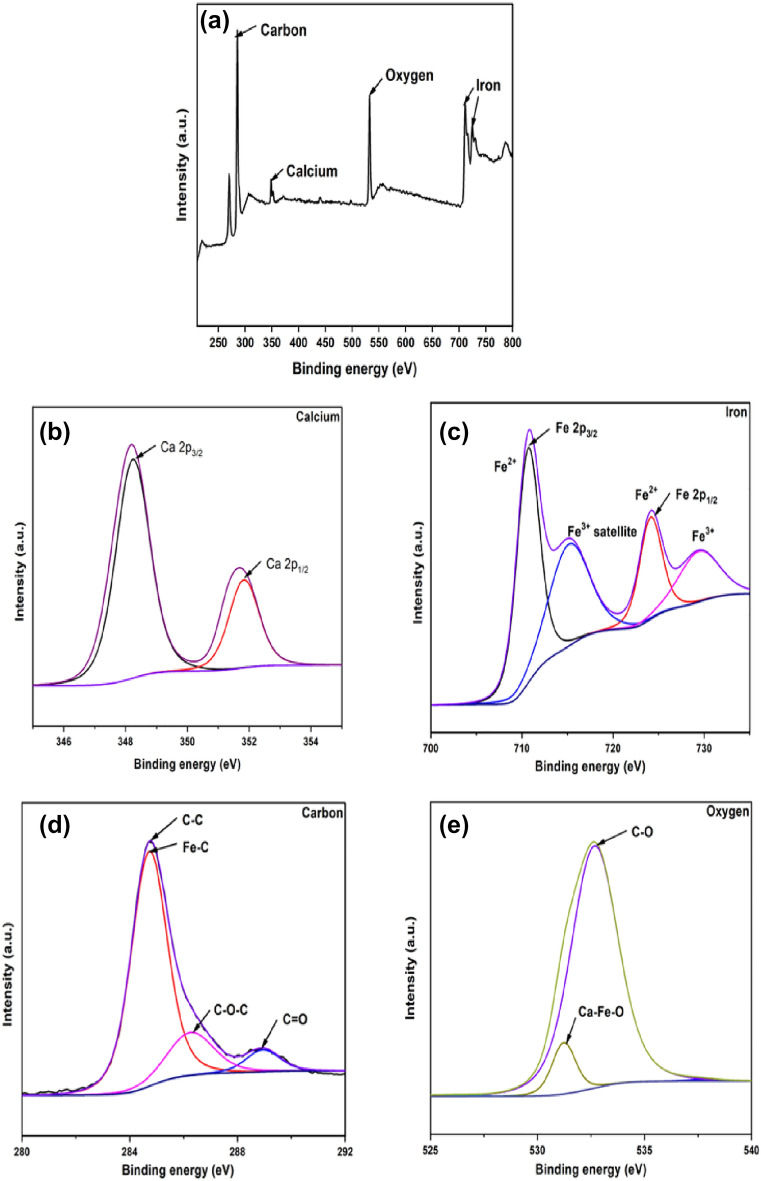
(**a**) Overall XPS spectra and deconvoluted spectra of (**b**) calcium, (**c**) iron, (**d**) carbon and (**e**) oxygen present in the CFB nanocomposite.

The valence state of the four components, such as calcium, iron, oxygen, and carbon, in the CFB nanocomposite was investigated using XPS analysis (Fig. 5a). Table 1 shows the full width at half maximum (FWHM), counts per second (CPS), atomic %, and sensitivity factor (SF) of components as obtained by XPS spectra.

**Table 1:** Data obtained from XPS spectra showing the presence of C, O, Fe and Ca.

Name	Peak BE	FWHM eV	Area CPS	Atomic %	SF
Carbon	285.22	2.98	1166769.35	71.54	1
Oxygen	532.77	3.25	708145.62	17.97	2.881
Iron	711.22	4.61	1418866.28	8.44	14.353
Calcium	348.32	2.66	191958.96	2.05	5.97

XPS spectra of the calcium ion reveal the two photoelectron lines. Both binding energy maxima, at 348.20 and 351.80 eV, correspond to orbitals in the calcium ions 2p_3/2_ and 2p_1/2_ shells, respectively, and show the presence of Ca^2+^ in the CFB nanocomposite^49^ (Fig. 5b). The deconvoluted XPS spectra of the Fe 2p atom show four photoelectron lines (Fig. 5c). Iron shows maximum peaks at 710.75 and 724.18 eV, which are related to Fe 2p_3/2_ and Fe 2p_1/2_, respectively, and confirm Fe^2+^ presence. The spectra display additional peaks at 715.38 and 729.58, which are satellite peaks of Fe^3+^ ions that are 4.6 and 5.4 eV above the photoelectron line^41^. The magnetic moment in the lattice is fundamentally set by the proportion of Fe^2+^ ions present^50^. The measured spectrum reveals the presence of Fe^3+^ ions in the CFB nanocomposite without the reduction of corresponding Fe^2+^ ions. These data show the simultaneous existence of Fe^2+^ and Fe^3+^ in the nanocomposite, showing the formation of Ca_4_Fe_9_O_17_. Carbon peaks at 284.74, 286.31 and 288.8 eV correspond to C–C/Fe–C, C–O–C, and C=O, respectively (Fig. 5d)^51,52^. The O 1s XPS spectra showed two photoelectron lines, one at around 531.15 eV and the other at approximately 532.66 eV (Fig. 5e). The 531.15 eV component is attributable to O_2_^–^ ions bound to Ca^2+^ and Fe^3+^^53^. In contrast, the peak at higher maxima corresponds to C-O bonds obtained from biochar. The XPS data proved that Ca_4_Fe_9_O_17_ was successfully incorporated with biochar. All these data fairly matched with the XRD, FTIR, and HR-TEM analysis which describes the successful formation of CFB nanocomposite.

### Raman and FTIR spectra

**Figure 6:**
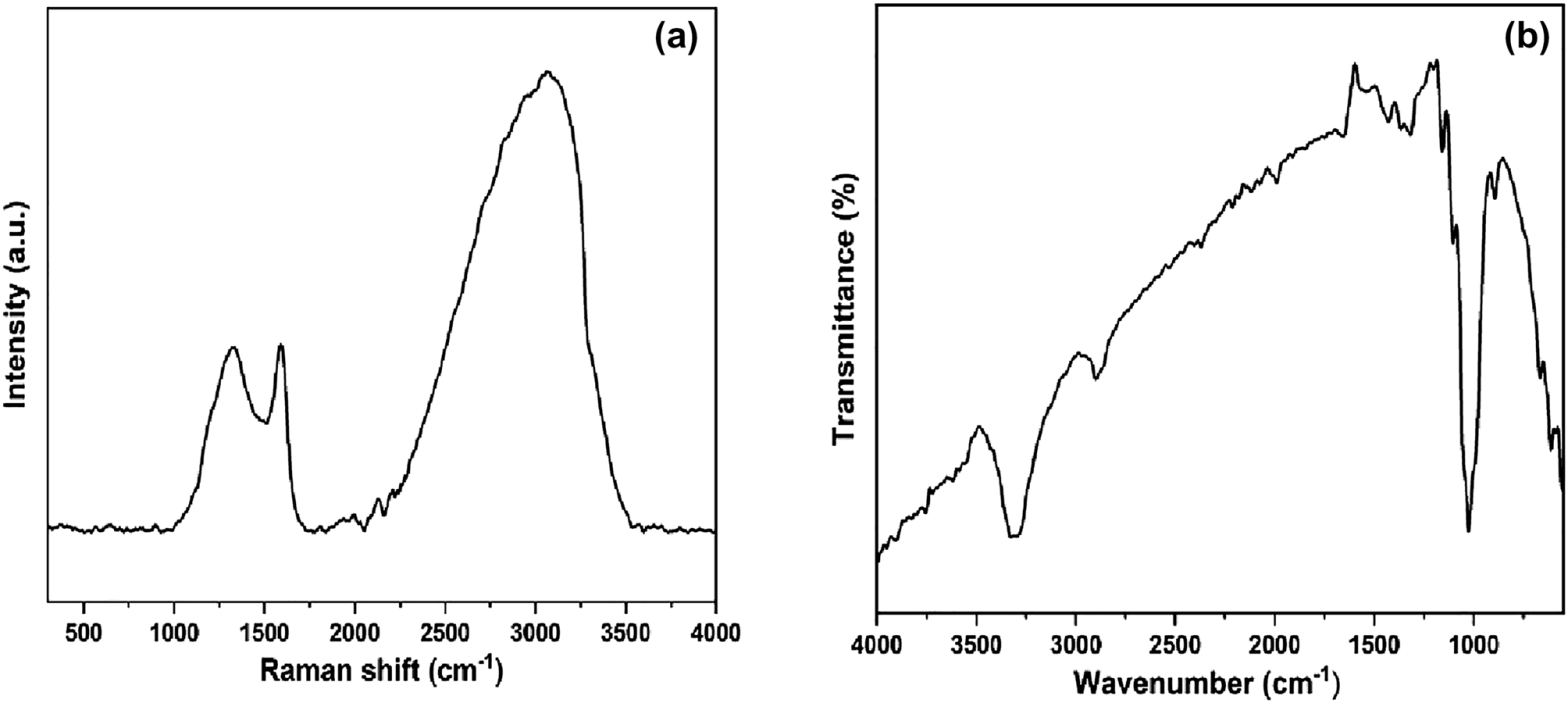
(**a**) Raman spectra and (**b**) FTIR spectra of the CFB nanocomposite.

The disorganized architecture of CFB nanocomposite was studied in more detail using Raman analysis. The spectral lines of CFB split into three distinct peaks at 1320 cm^–1^, 1586 cm^–1^, and 3068 cm^–1^, (Fig. 6a), which correspond to disordered carbon, graphitic carbon, and aromatic carbon, respectively^54^. Higher pyrolysis temperature leads to an increase in the disorder of the CFB nanocomposite, resulting in an increase in the reactive sites to catalyze H_2_O_2_^55^. The chemical bonding and chemical composition of CFB nanocomposite was determined by FTIR spectroscopy (Fig. 6b). FTIR analysis was performed at room temperature in the range of 4000–550 cm^–1^. Multiple peaks in the 3500^–^600 cm^–1^ range represent the presence of several functional groups. The O–H stretching was responsible for a broad peak at 3311 cm^–1^ in the FTIR spectra obtained due to the H_2_O group engaged in hydrogen bonding^56^. A narrow peak near 1563 cm^–1^ and 1648 attributed to C=C and C=O stretching in the biochar^57^. The C-H stretching bonds are responsible for the absorption peaks at 2905 cm^–1^. The distinct peak at 1155.04 cm^–1^ corresponds to the C–O stretching bond. The stronger peak at 894 cm^–1^ was attributed to the Fe–O bending vibrations in the CFB nanocomposite^58^, while the C–C bending vibrations were responsible for the weaker peak of 616.21 cm^–1^. Due to the presence of the spinal ferrite skeleton, the bands at 667 cm^–1^ are ascribed to Fe–O bonds, whereas the band at 1028 cm^–1^ is associated with metal alloy (Fe-Ca)^59^. Therefore, the data of the FTIR fairly shows the formation of CFB nanocomposite.

### AOP-assisted degradation of dyes using CFB nanocomposites

Various significant factors influence the efficiency of AOP and the removal of pollutants from wastewater. In this section, we will illustrate the effect of H_2_O_2_ concentration, catalyst loading, pollutants concentration, and contact time on the AOP-assisted degradation of dyes.

#### H_2_O_2_ concentration

H_2_O_2_-assisted AOP reactions are regarded as promising techniques for pollutant degradation due to the formation of highly reactive oxygen species (ROS) named hydroxyl radicals and superoxide radicals. Pristine CFB nanocomposite showed 4.43 and 5.12% removal of RhB and MB in 60 min, respectively, demonstrating that the catalyst’s adsorption properties were insufficient for the removal of pollutants. Hydrogen peroxide was used as an oxidizing agent, and the removal efficiency of MB and RhB increased by increasing H_2_O_2_ concentration from 0.3 mL to 0.9 and 1.2 mL (Fig. 8a,b). About 98.5% removal of MB and 95.32% removal of RhB could be achieved at an H_2_O_2_ dosage of 0.9 mL and 1.2 mL, respectively. The increased efficiency was attributed to the production of more active species as a result of increased H_2_O_2_ addition. Surface redox cycling of Fe^2+^ and Fe^3+^ at the CFB nanocomposite has a role in the reaction, and ferrous species catalyze the oxidants to create additional reactive radicals (Eqs. 5, 6)^60^.

5 $${\text{Fe}}^{2 + } + {\text{ H}}_{2} {\text{O}}_{2} \to {\text{Fe}}^{3 + } + {\text{ OH}}^{ - } \, + {\text{ OH}}^{ \cdot } ,$$

6 $${\text{Fe}}^{{{3} + }} + {\text{H}}_{{2}} {\text{O}}_{{2}} \to {\text{Fe}}^{{{2} + }} + {\text{ HO}}_{{2}}^{ \cdot } + {\text{ H}}^{ + } .$$

The diffused ferrous ions in the solution trigger the decomposition reaction of H_2_O_2_. Finally, reactive species generated in the solution phase and the catalyst surface initiate a series of reactions causing the degradation of the pollutants. The oxidative degradation rate is significantly enhanced due to H_2_O_2_’s preferential reaction with the ferrite phases in the CFB nanocomposite^61^. A decrease in the degradation efficiency was observed on further increasing the concentration of H_2_O_2_, which may be due to competition from other radicals for available active sites^62^. Another factor that contributed to the efficiency decline was found to be the scavenging of OH^·^ at higher concentrations of H_2_O_2_ according to the following equation (Eq. 7):

7 $${\text{H}}_{2} {\text{O}}_{2} + {\text{ OH}}^{ \cdot } \to {\text{HO}}_{2}^{ \cdot } + {\text{ H}}_{2} {\text{O}}.$$

**Figure 7:**
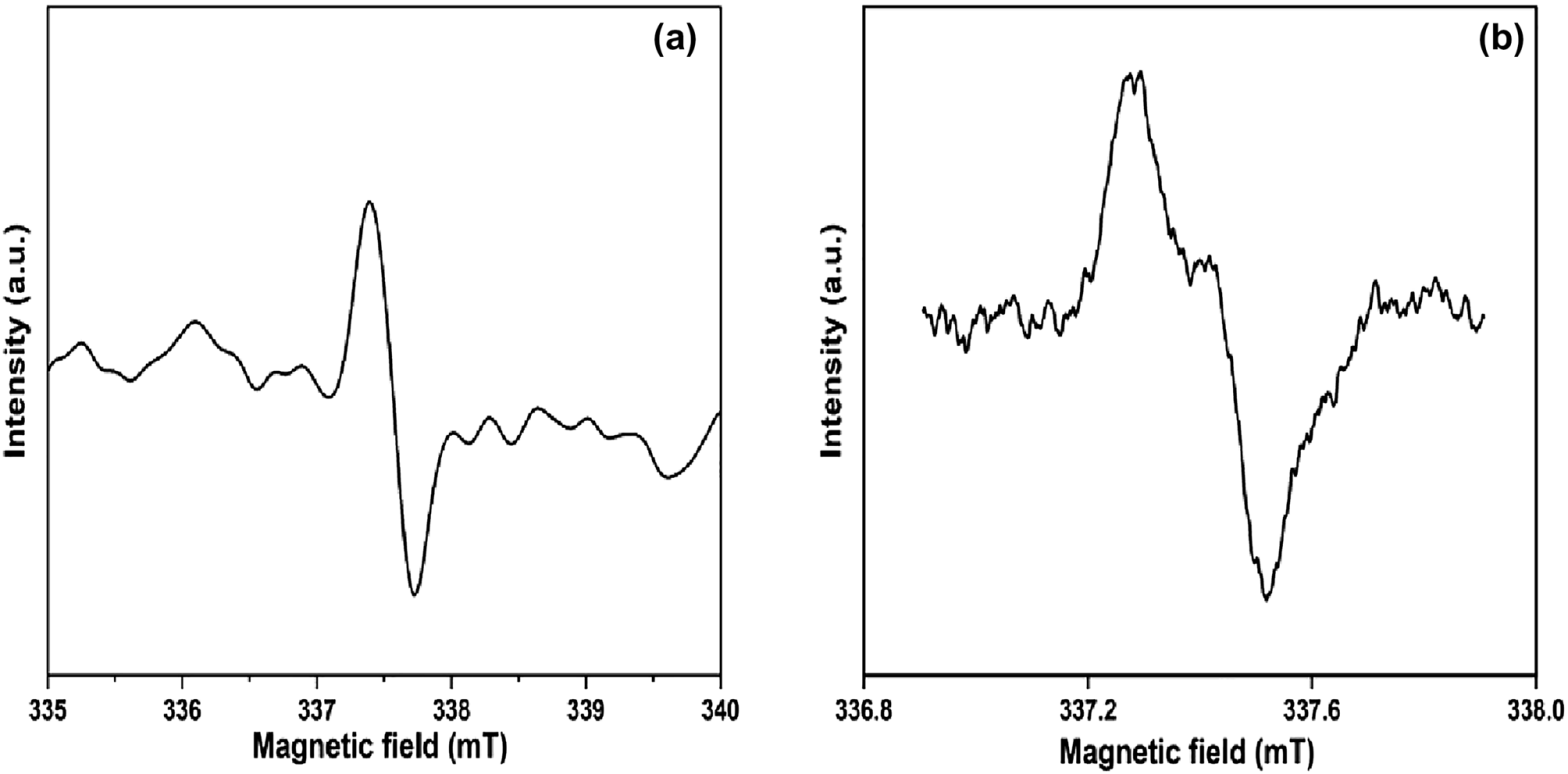
Electron spin resonance (ESR) of reactive (**a**) hydroxyl, (**b**) superoxide radicals.

Reactive oxygen species generated during the course of the reaction were analyzed by electron spin resonance in the reaction system produced by CFB nanocomposite. It was clear from the ESR spectra that TEMPO-OH^·^ and TEMPO-O_2_^·^¯ radicals are the main reactive species, as seen in Fig. 7a,b. These data explain the radical pathway resulting in the degradation of pollutants.

#### Catalyst loading

**Figure 8:**
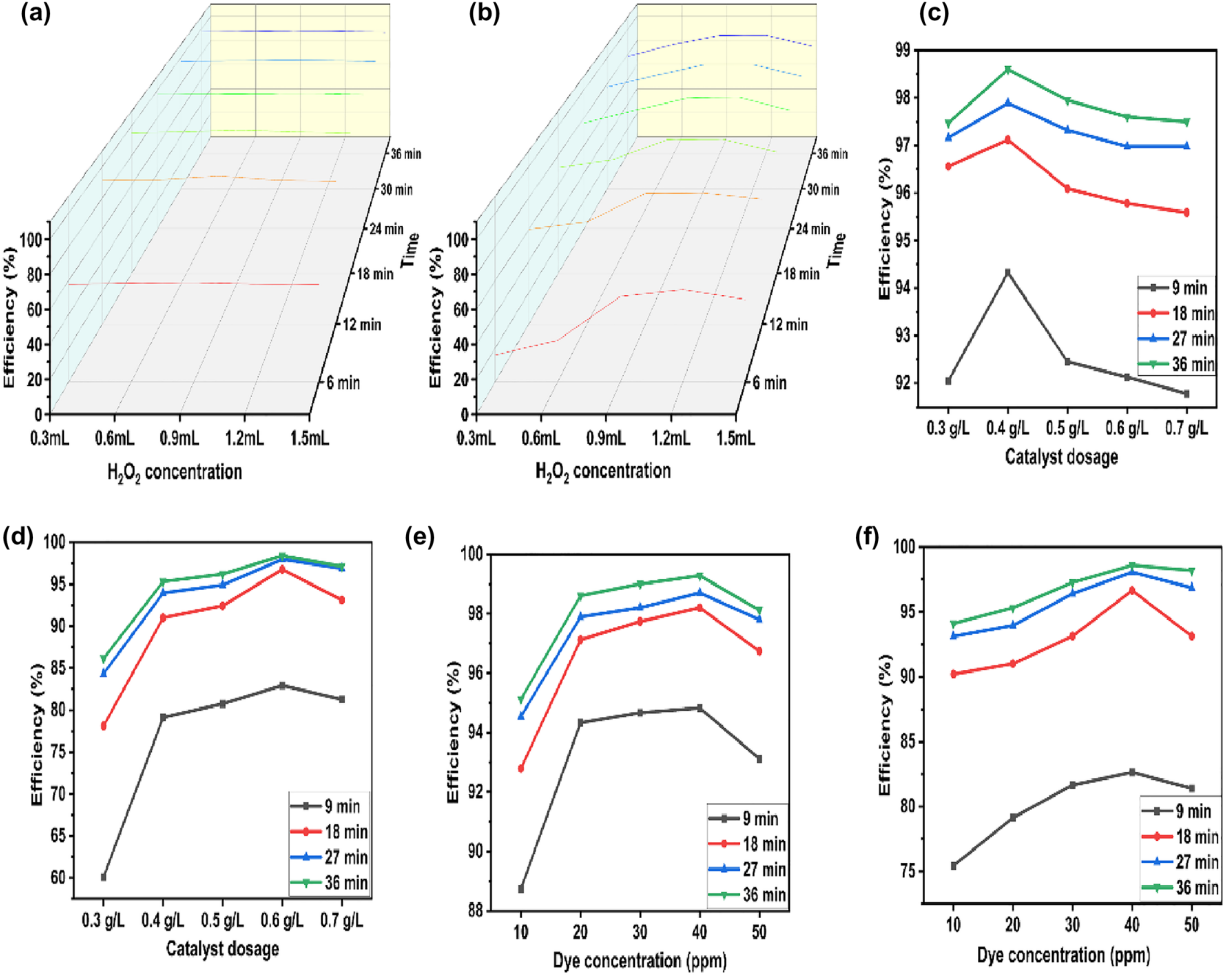
Influence of various parameters on dyes degradation H_2_O_2_ optimization on (**a**) MB (**b**) RhB, Catalyst dosage on (**c**) MB (**d**) RhB, Dye concentration on (**e**) MB (**f**) RhB.

In order to determine the efficiency of the catalyst (CFB nanocomposite), the catalyst dosage was varied from 0.3 to 0.7 g/L by keeping all other variables constant. The H_2_O_2_+CFB system showed enhanced removal of dyes compared to only H_2_O_2_ and pristine CFB, demonstrating that the CFB nanocomposite is a potential activator of H_2_O_2_ for pollutant removal. A graph depicting the degradation efficiency of pollutants in the presence of varying dosages of CFB nanocomposite is shown in Fig. 8a,b. As expected, the degradation efficiency increased with increasing catalyst loading (Fig. 8c,d). The results showed that about 98.4% of MB and 97.4% of RhB could be degraded by 0.4 g/L and 0.6 g/L of catalyst loading, respectively. The increased efficiency was attributed to an increase in the number of active sites on a catalyst’s surface, which attracted the pollutant molecules from the solution. However, at higher loading, the formation of excessive radicals led to the quenching of the removal process^63^. Therefore, the optimum catalyst loading for MB and RhB was found to be 0.4 g/L and 0.6 g/L, respectively.

#### Initial concentration

The initial concentration of dyes is also an essential factor, which affects the catalytic activity of the nanocomposite. Experiments were conducted with the optimum conditions (H_2_O_2_ concentration and catalyst loading) and varying concentrations of dyes (10 ppm to 50 ppm) to analyze the way in which the concentration of dyes impacts the degradation process. Figure 8e,f shows the dye concentration graph. Unexpectedly, the degradation efficiency was found to increase with the concentration of the dyes. Maximum degradation of 99.2% of MB and 98.6% of RhB could be achieved under optimum conditions for 40 ppm of dyes. The degradation efficiency directly correlates with the number of reactive oxygen species generated and their interaction with the pollutants. Thus, the optimal concentration for both dyes (MB and RhB) was found to be 40 ppm. Also, the catalyst works well for higher concentrations of dyes; however, a slight decrease in the removal efficiency was observed with a further increase in the concentration of dyes. The reduction in efficiency may be due to the adsorption of dye molecules on the catalyst surface. This caused the catalyst to produce insufficient amounts of superoxide anion radicals (O_2_^·^¯) and hydroxyl radicals (OH^·^)^64^. At higher concentrations of dyes, there was self-quenching of radicals with dye molecules occurs, which resists the degradation of dyes molecules.

#### Contact time

**Figure 9:**
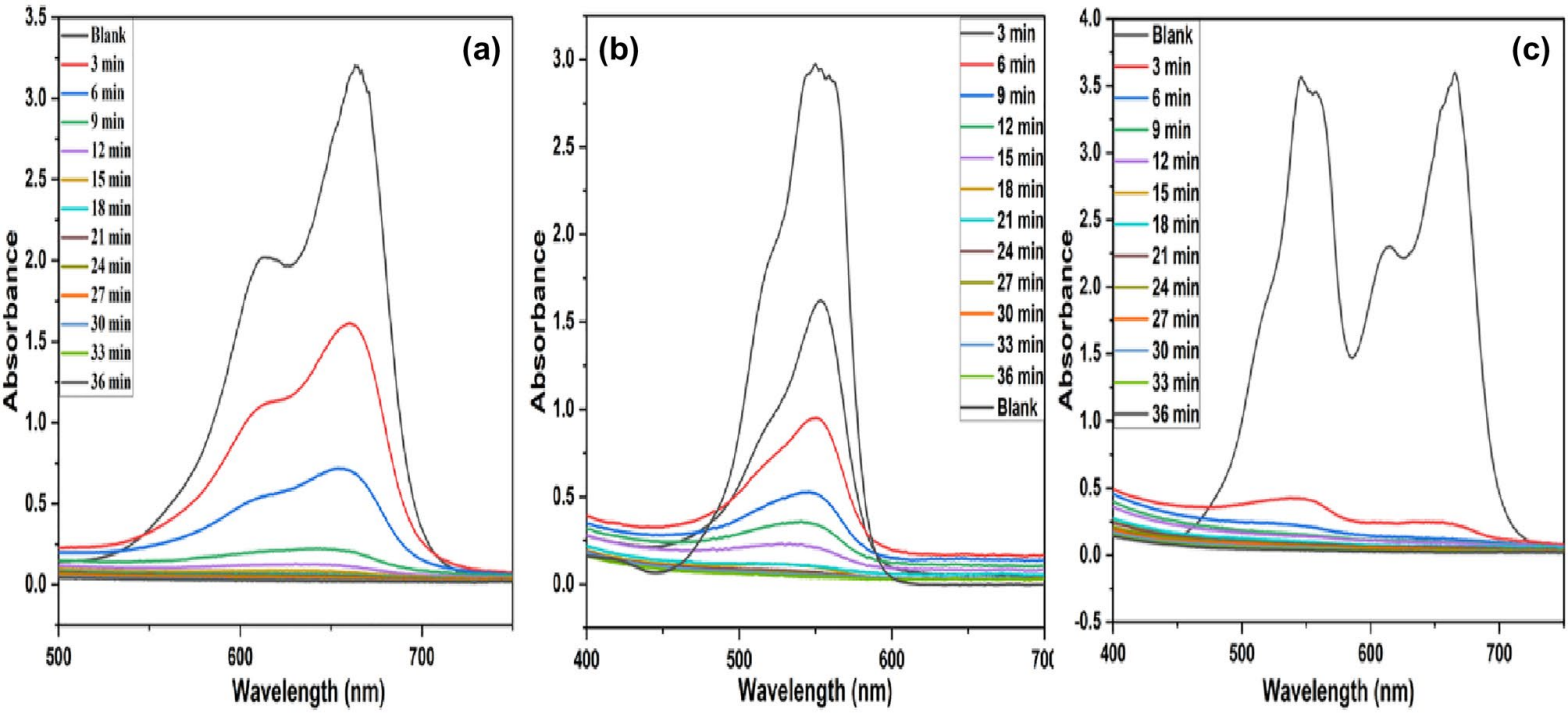
Contact time graph for (**a**) MB dye, (**b**) RhB dye and (**c**) Mixed MB and RhB dyes.

Under the optimal H_2_O_2_ concentration, catalyst dosage, and dye concentration, the effect of contact time had been studied at different time intervals. The catalyst loading of 0.4 g/L and 0.6 g/L for MB and RhB, respectively, was added to 40 ppm of dye concentration, and the resulting solution was kept for 36 min. The maximum degradation of 99.28% and 98.6% for MB and RhB, respectively, was observed under a short span of 36 min (Fig. 9a,b). Furthermore, no appreciable increase in the degradation efficiency was observed after 36 min, which could be due to the exhaustion of the surface active sites of the catalyst or may be due to the adsorption of intermediate products on the biochar surface^65^.

### Reaction kinetics

**Figure 10:**
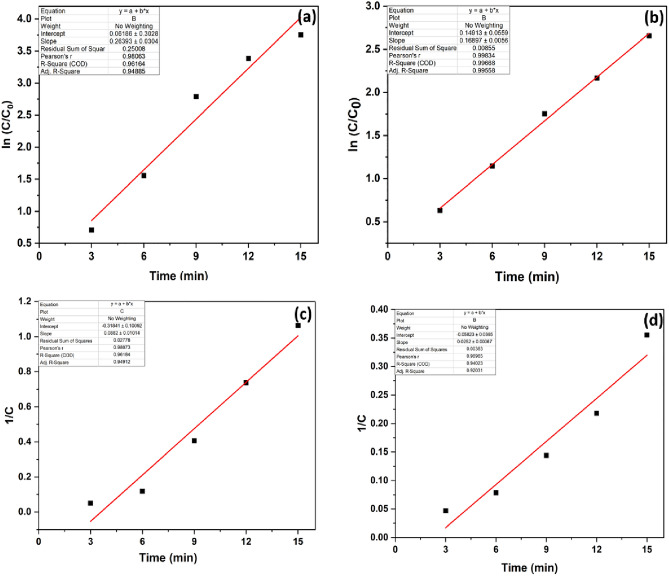
Pseudo 1st order kinetics of (**a**) MB and (**b**) RhB dyes and pseudo 2nd order kinetics of (**c**) MB and (**d**) RhB.

The kinetics of MB and RhB degradation over time was shown in Fig. 10. MB and RhB degradation follow pseudo-first-order kinetics with a rate constant of 0.2639 and 0.168 min^–1^ for MB and RhB, respectively, as seen by the logarithmic plot of concentration against time for both dyes (Fig. 10a,b). The R^2^ for the pseudo-first-order reaction was 0.9616, and RhB was 0.9966. Whereas the pseudo-second-order rate constant for MB and RhB was calculated to be 0.0882 and 0.0252 min^–1^, with R^2^ values of 0.96 and 0.94, respectively.

### Mixed dyes

Only a few researchers were investigating the potential of a catalyst in a multipollutant system. Therefore, the applicability of the CFB nanocomposite for the enhanced degradation of a binary mixture of the dyes was also investigated for environmental remediation. To further evaluate the synergistic effect of dyes on the catalytic activity of the CFB nanocomposite, an equivalent mixture of both the dyes (MB and RhB) was taken. It is worth mentioning that no change in the dye concentration was observed in the absence of CFB nanocomposite, suggesting that the dyes are resistant to self-oxidation. Initially, the concentration of H_2_O_2_ was optimized for the degradation of the binary mixture of dyes using 50 mL of 20 ppm solution of each dye. The optimum H_2_O_2_ concentration was observed to be 0.3 mL of the mixture of dyes, which was significantly lower in comparison to single dye {MB (0.9 mL) and RhB (1.2 mL)}. This change may be due to the synergistic effect between dyes in charge delocalization and enhanced charge transfer by the catalyst^66^. The catalyst dose optimization followed the same procedure as mentioned in the above section. The optimal catalyst dosage for the mixture of dyes was found to be 0.4 g/L. Interestingly, an increase in the degradation efficiency of the dyes was observed compared to a single dye system. Under optimum conditions, near to about complete degradation of both dyes was observed within 36 min. The degradation efficiency for RhB and MB reached up to 99.2 and 99.4% in the dye mixture as compared to individual (98.6% for RhB and 99.2% for MB), respectively, in the same period, when the initial concentration of 20 ppm (20 ppm MB and 20 ppm RhB) dyes was taken with a catalyst loading of 0.4 g/L. Absorption maxima of MB at 663 nm and RhB at 551 nm, as shown in Fig. 9c, slumped with continuing time and disappeared after 36 min in the presence of CFB nanocomposite. It was noticeable from the contact time graph that the absorption intensity of binary dye solution decreased significantly as compared to single dye. These results indicate that CFB nanocomposite is a viable source for the environmental remediation of individual and mixed dye solutions. Similar types of studies were done by many researchers and reached the same conclusion^67–69^. Table 2 shows the comparative study of the mixed dyes of the present investigation with the reported values in the literature.

**Table 2:** Comparison of this study with the previously reported study of various mixed dye solution.

Catalyst	Pollutants	Catalyst dosage (g/L)	Initial concentration	Efficiency (%)	Time (min)	References
Au-ZnO	MB and RhB	–	60 µM	98, –	120	^70^
ZnFe_2_O_4_@g-C_3_N_4_	MB and RhB	1	20 ppm	92, 100	35	^71^
Bi_2_S_3_	MB and RhB	2	20 ppm	52, 60	150	^66^
Sn doped BiOCl	RhB and MO	0.5	20 ppm	99, 93	480	^72^
BiOCl	MO and RhB	0.6	1.5 µM and 5 µM	–	120	^36^
CFB	MB and RhB	0.4	40 ppm at pH 7	99.4, 99.2	36 min	This study

### LC-MS analysis

**Figure 11:**
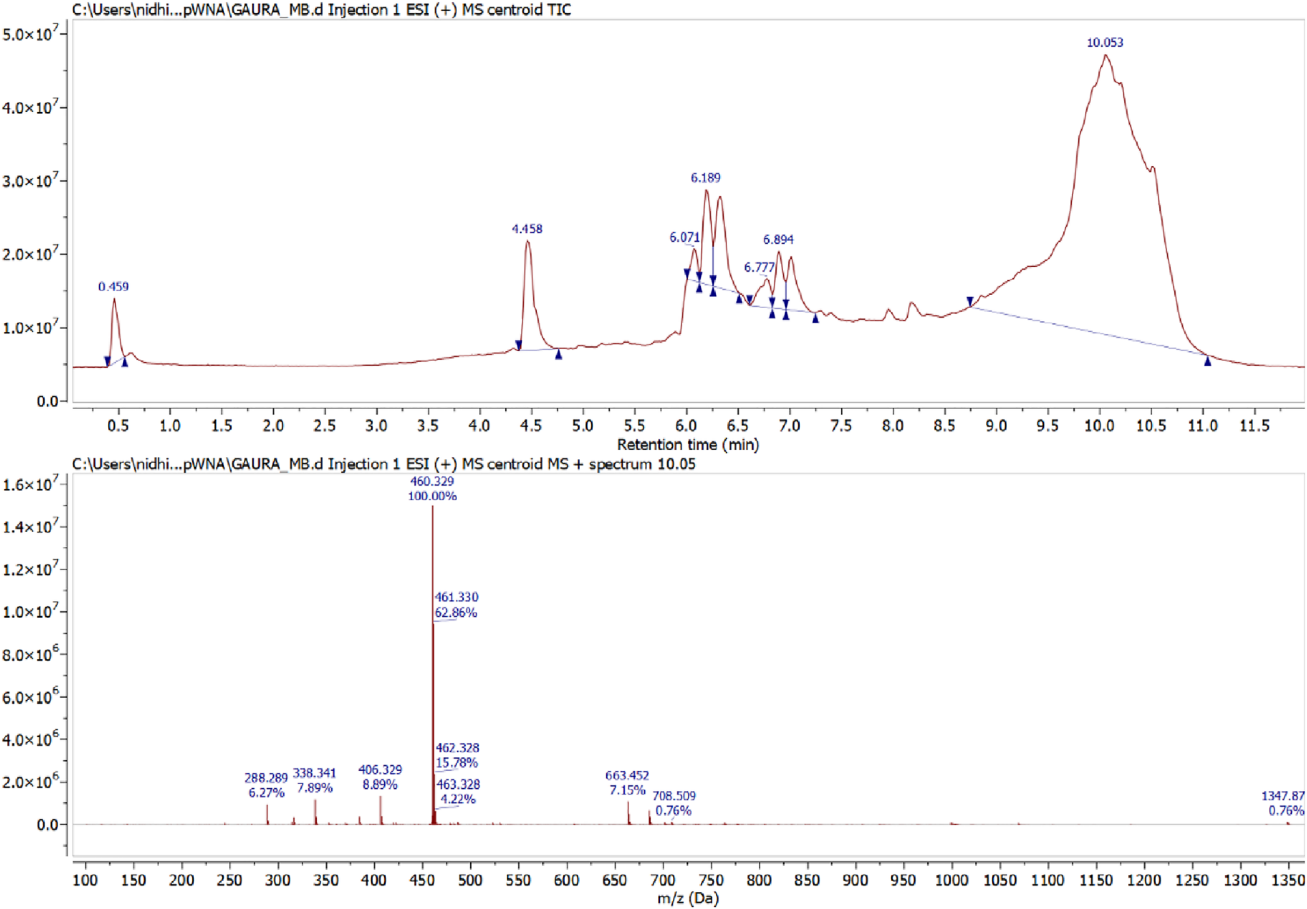
HPLC with LC-MS data of MB.

**Figure 12:**
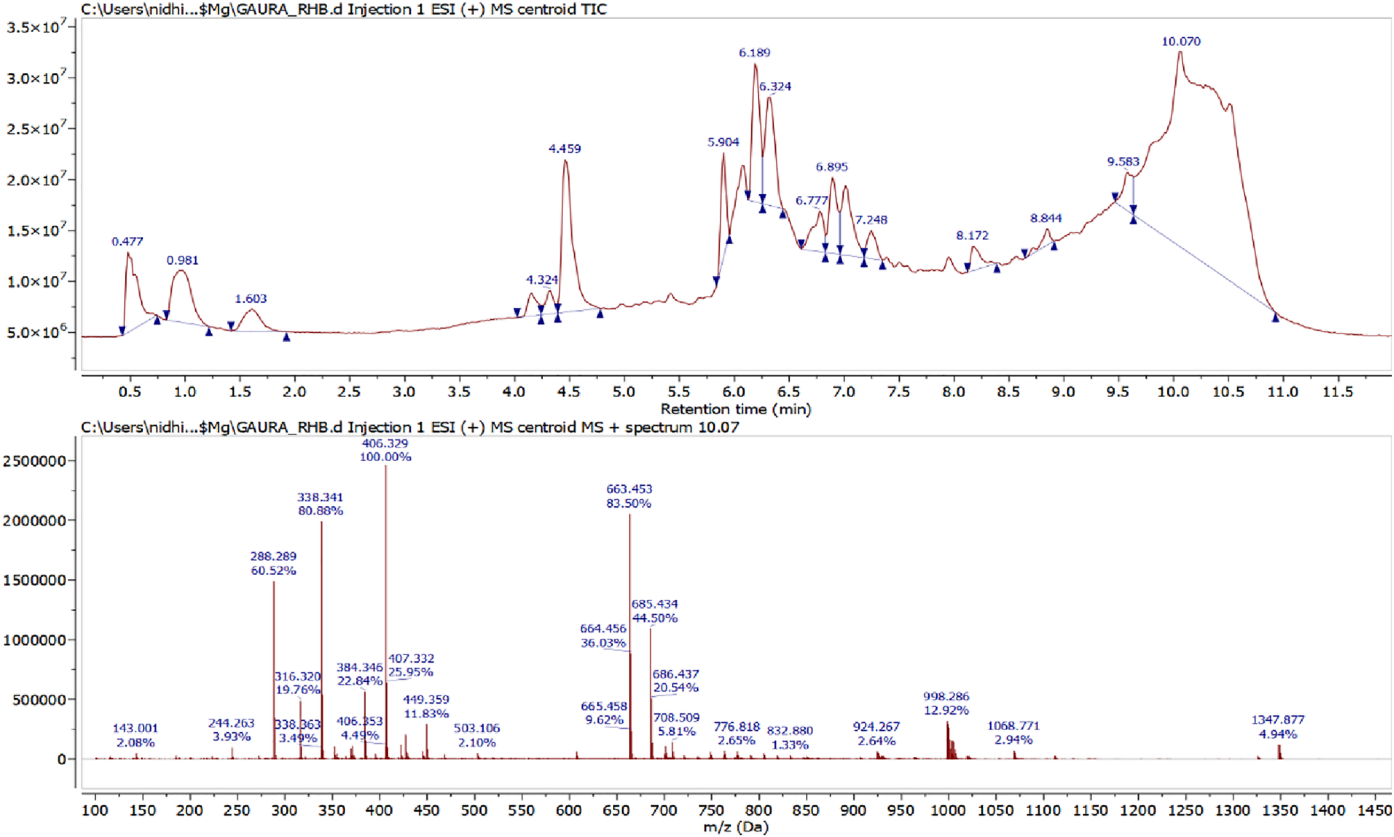
HPLC with LC-MS data of RhB.

**Figure 13:**
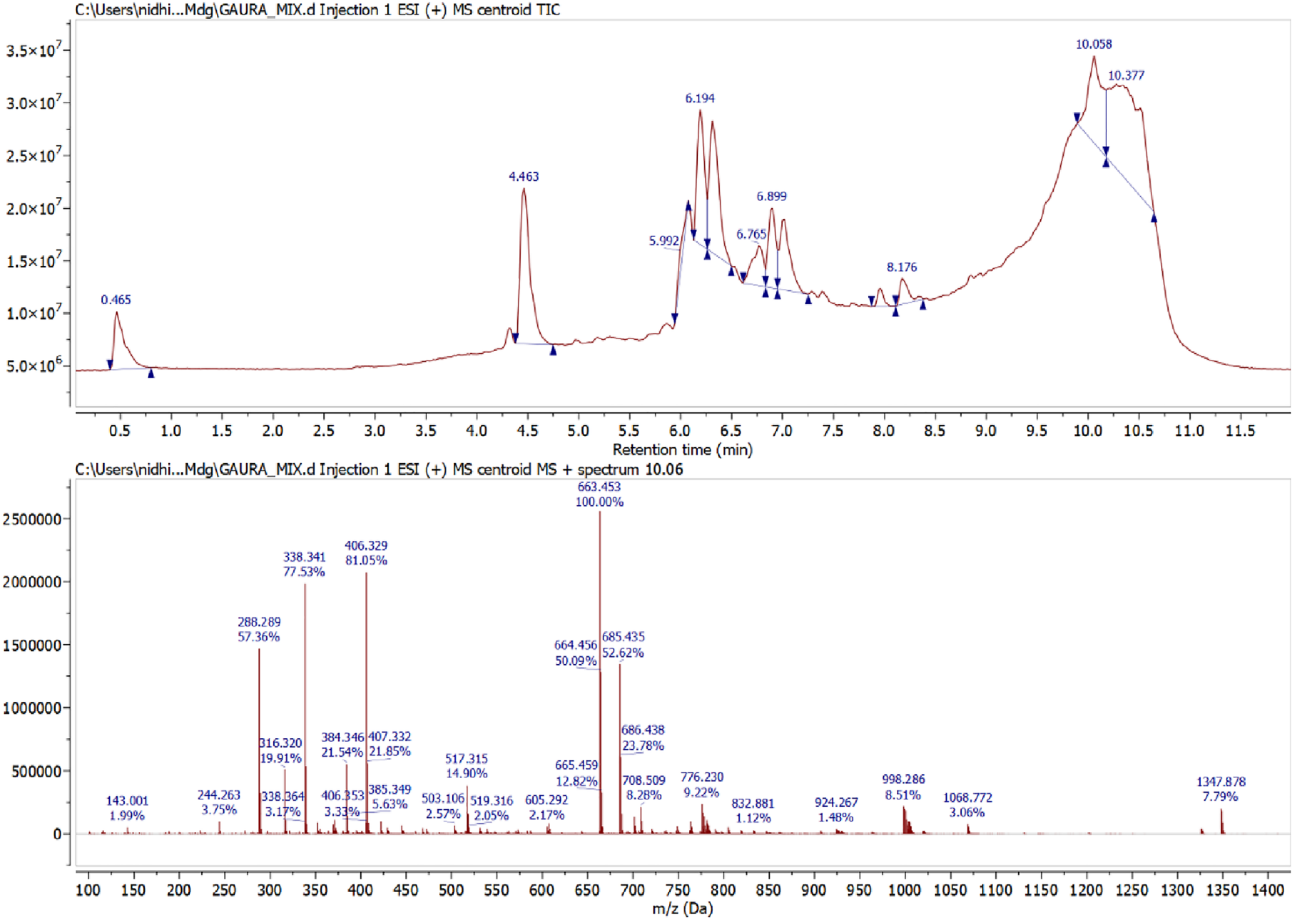
HPLC with LC-MS data of binary dye solution.

**Figure 14:**
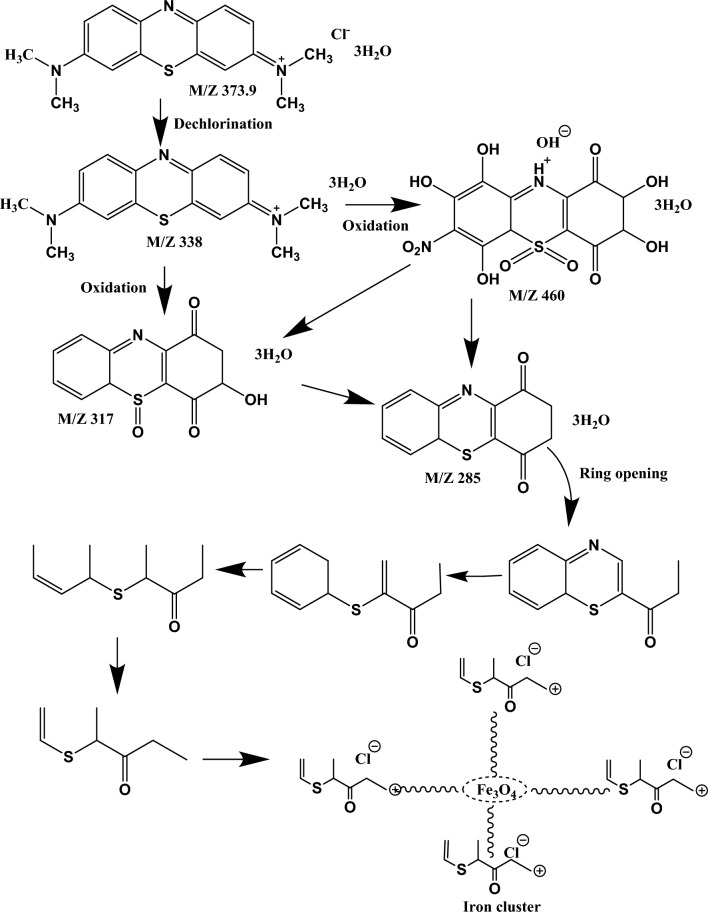
Possible degradation pathway of MB and formation of the iron cluster.

**Figure 15:**
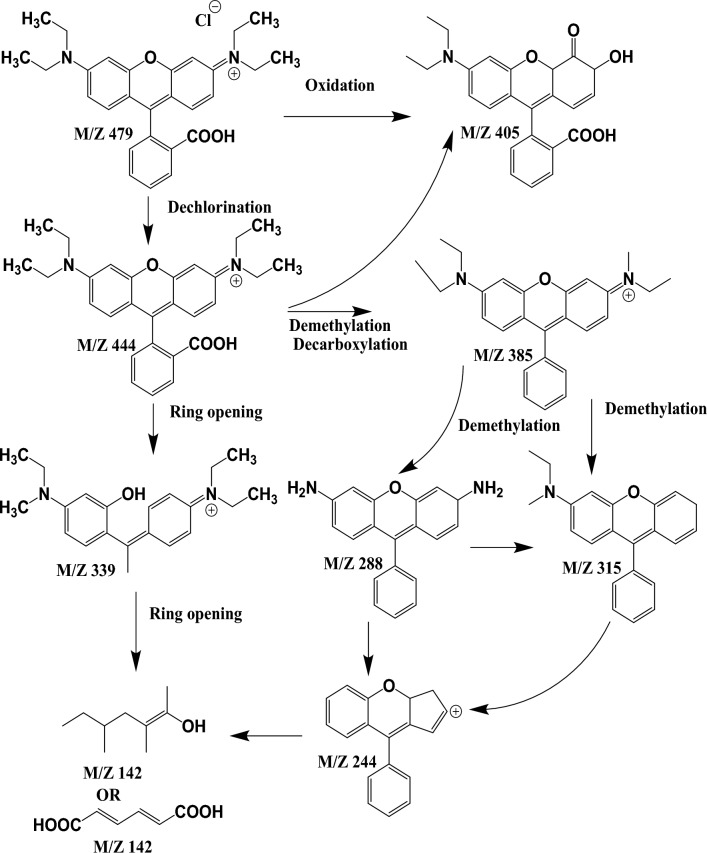
Possible degradation pathway of RhB degradation.

The final degraded dye solution was characterized by LC-MS to examine the end product and the degradation pathway. The mass spectra of ions generated during the AOP process were shown in (Figs. 11, 12 and 13). As seen in Fig. 11, the prominent peak of MB was shown at m/z 460, which indicates that MB dye undergoes the oxidation process. In contrast, RhB dye shows significant peak intensity at m/z 406 (Fig. 12). Whereas mix dye LC-MS data indicate the absence of a substantial peak of MB at m/z 460, it can be concluded from the LC-MS data that no MB oxidation occurs during binary solution mixture (Fig. 13). Dechlorination was the 1st step of the degradation of MB and RhB. As seen in Fig. 14, after the dechlorination step, oxidation of MB occurs, followed by ring opening. Whereas RhB follows the demethylation, decarboxylation, and finally, ring opening takes place. Figs. 14 and 15 show the probable mechanistic degradation pathways of the degradation of dyes.

### Mechanism study of dyes degradation

**Figure 16:**
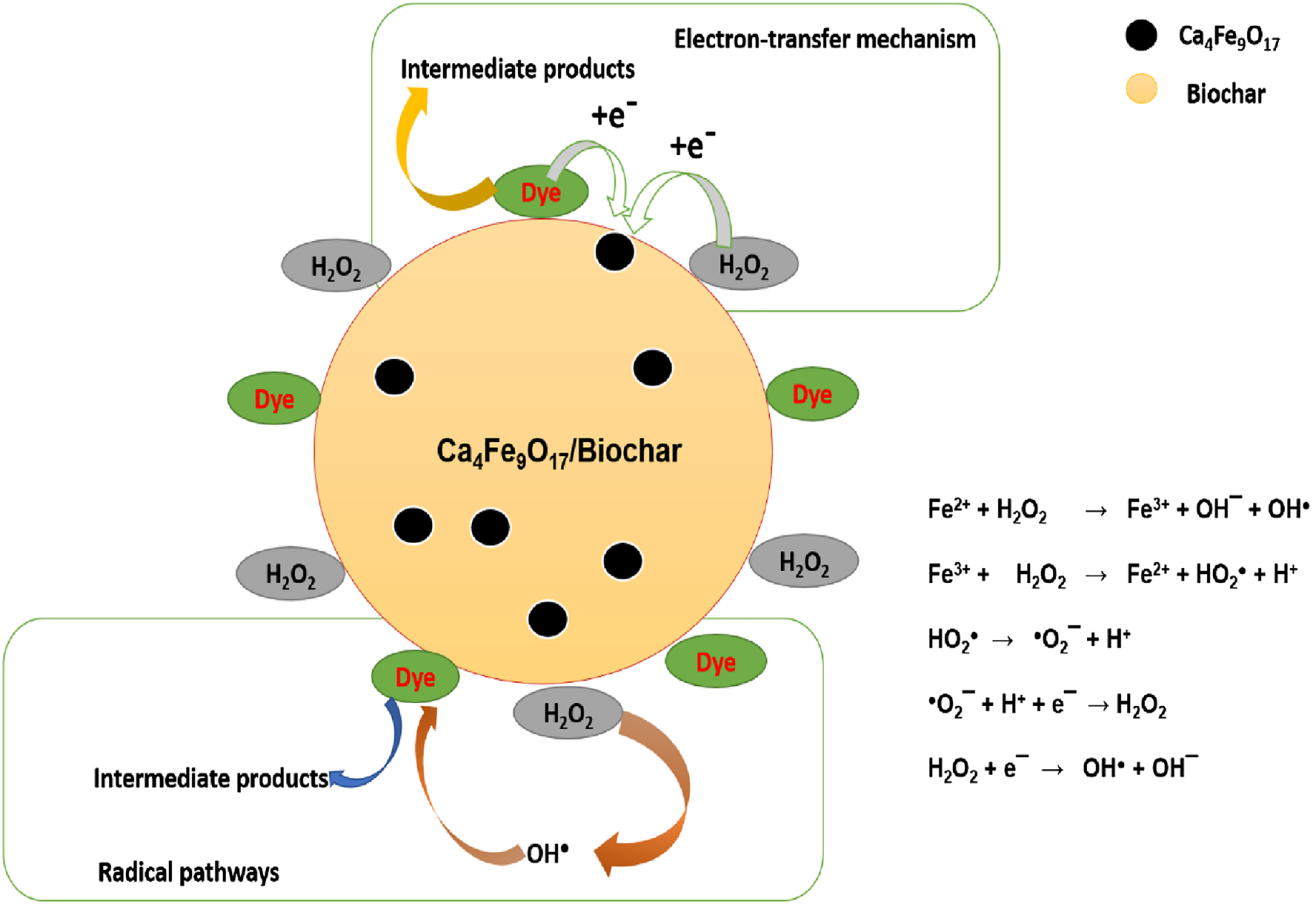
Possible reaction mechanism of CFB nanocomposite for dye degradation.

Hydrogen peroxide is a potent oxidizing agent having a redox potential of 1.8 eV. However, its restricted reaction rate limits itself to oxidizing pollutants. Studies show that the removal of dyes occurs either by radical or electron transfer method in the H_2_O_2_ activation process. Iron plays a critical role which provides active sites and helps in easy separation. Fe^3+^ regenerates Fe^2+^ in the presence of H_2_O_2_ and forms OH^·^, additionally accelerating the oxidation of pollutants. The ESR study demonstrated the radical species found in the reaction mixture. ESR shows that hydroxyl and superoxide radicals were the dominant species in the reaction mixture which helps in dye removal. Adsorption on CFB was significantly aided by its remarkable features, such as its numerous functional groups, highly disordered structures, high SSA, and evenly dispersed Ca_4_Fe_9_O_17_ particles. As a result of this adsorption, the contact area and the mass transfer rate between the pollutants and free radicals were enhanced. During the first phase of the process, CFB's nanocomposite surface was covered with molecules of H_2_O_2_ and dyes, resulting in the generation of free radicals, which oxidize the dye molecules (Eqs. 8, 9, 10, 11, 12 and 13). The activation of H_2_O_2_ by CFB nanocomposite (active sites such as Fe (II), C=O functional groups, and defects) responsible for the production of OH^·^ and O_2_^·^¯. In conclusion, the degradation of MB or RHB dye might exhibit direct electron transfer or radical route (Fig. 16). There is more possibility that reaction pathways occur by radical route. This mechanism relates to the previously published literature^73,74^.

8 $${\text{Fe}}^{2 + } + {\text{ H}}_{2} {\text{O}}_{2} \to {\text{Fe}}^{3 + } + {\text{ OH}}^{ - } \, + {\text{ OH}}^{ \cdot } ,$$

9 $${\text{Fe}}^{{{3} + }} + {\text{H}}_{{2}} {\text{O}}_{{2}} \to {\text{Fe}}^{{{2} + }} + {\text{ HO}}_{{2}}^{ \cdot } + {\text{ H}}^{ + } ,$$

10 $${\text{HO}}_{2}^{ \cdot } \to ^{ \cdot } {\text{O}}_{2}^{ - } \, + {\text{ H}}^{ + } ,$$

11 $${\text{O}}_{{2}}^{ \cdot - } \, + {\text{ H}}^{ + } + {\text{ e}}^{ - } \to {\text{ H}}_{{2}} {\text{O}}_{{2}} ,$$

12 $${\text{H}}_{{2}} {\text{O}}_{{2}} + {\text{ e}}^{ - } \, \to {\text{OH}}^{ \cdot } + {\text{ OH}}^{ - } ,$$

13 $${\text{OH}}^{ \cdot } + {\text{ O}}_{{2}}^{ \cdot - } \, + {\text{ Dyes }} \to {\text{ Intermediate products }} \to {\text{ CO}}_{{2}} + {\text{ H}}_{{2}} {\text{O}}{.}$$

### Formation of iron cluster

ESR studies indicated the formation of superoxide and hydroxyl radicals due to the activity of CFB nanocomposite catalysts with H_2_O_2_, and these reactive oxygen species caused the degradation of MB and RhB dye as analyzed by LC-MS data. The reported fragments are shown in Figs. 11, 12 and 13. The LC-MS data show a peak of around m/z 285 for MB, which further degraded into a smaller compound to form an iron cluster. Additionally, EDS, XRD, and FTIR spectroscopy (Fig. 18) have all suggested that the other intermediate products created prefer to interact with iron to give non-cyclic/aliphatic iron-based deposited products.

#### SEM-EDS images of the iron cluster

**Figure 17:**
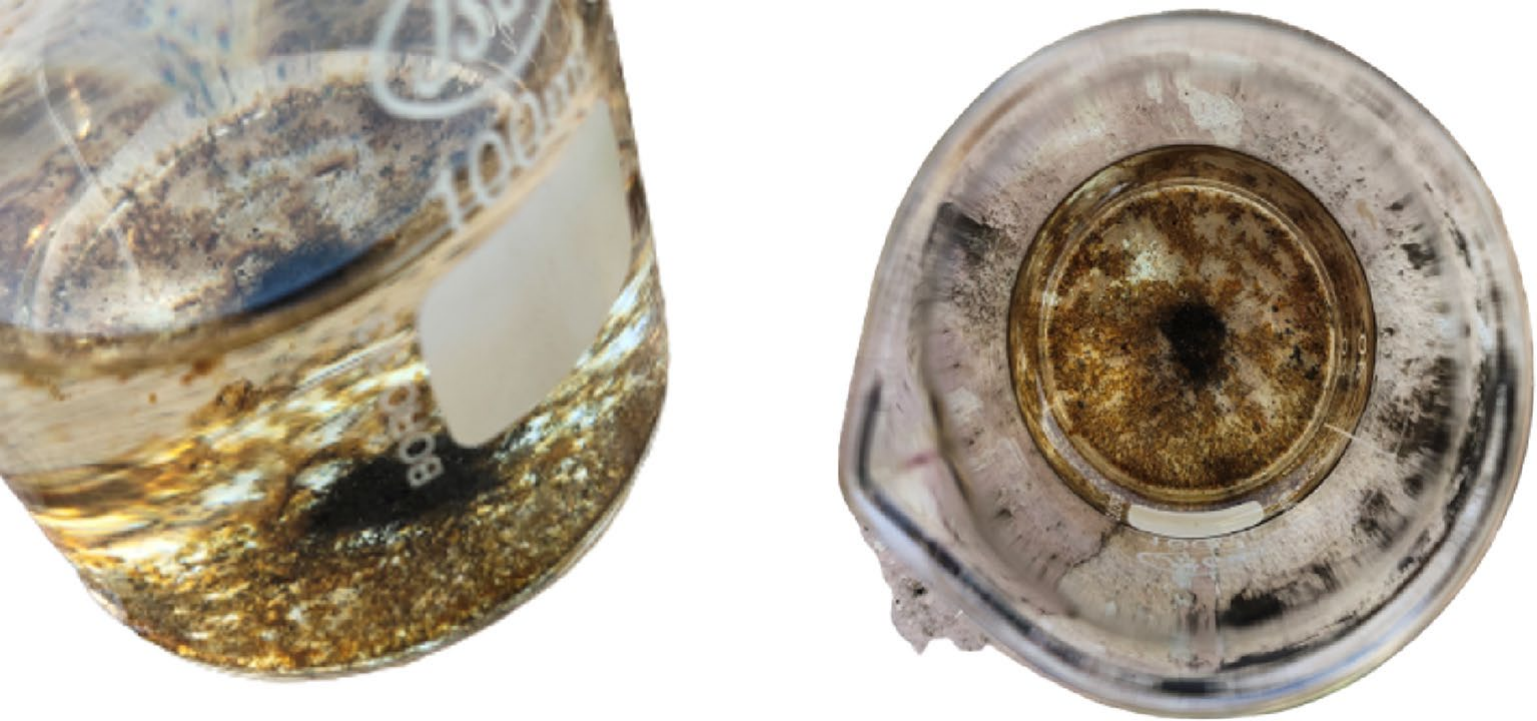
Formation of the iron cluster after dye degradation (MB).

**Figure 18:**
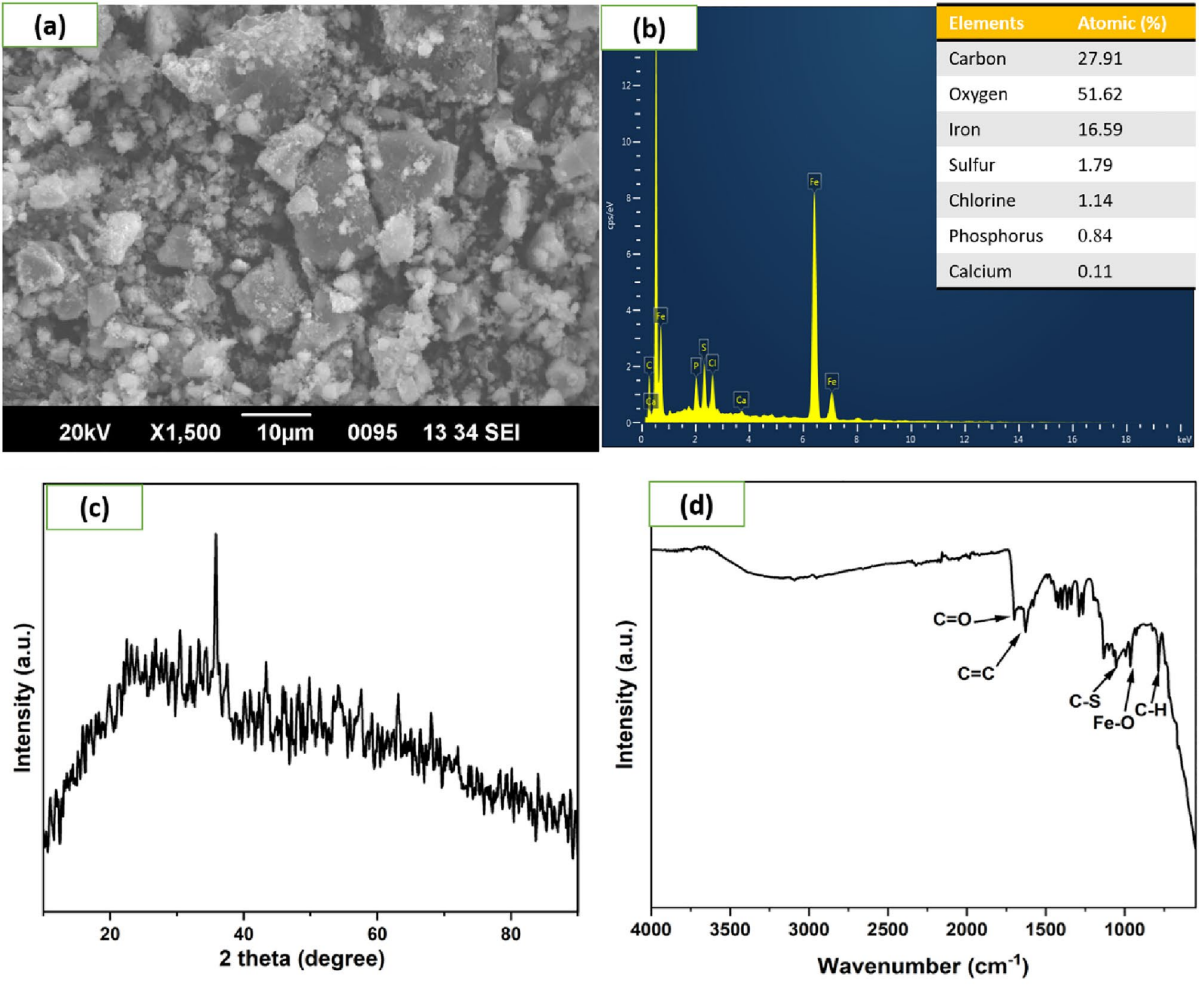
Iron cluster (**a**) SEM image, (**b**) EDS, (**c**) XRD, and (**d**) FTIR analysis.

SEM-EDS analysis displayed in Fig. 18 shows the morphology of the deposited product and its elemental compositions. The degraded dye left behind a deposited product (Fig. 17) with an uneven structure, as shown in the SEM picture (Fig. 18a). At the same time, the EDS analysis shows the presence of high concentrations of oxygen, carbon, sulfur, chlorine, and iron (Fig. 18b). This strongly suggests that the metal clusters seen in the treated solution were the byproduct of the breakdown of the dye. The sulfur content in the EDS analysis comes from the MB dye.

### XRD and FTIR spectra of iron cluster

The XRD pattern of the formed iron cluster from dye degradation was shown in Fig. 18c. The iron cluster formation starts after the degradation of pollutants and shows a prominent intense peak at 35.7°. The iron cluster peak matches with the Fe_3_O_4_ (JCPDS no. 880315). There is a slight shift in the intensity of the Fe_3_O_4_, indicating the formation of the iron cluster due to the presence of carbon, sulfur, and other atoms. It can be confirmed from the JCPDS that the product contains magnetite having a face-centered lattice and space group Fd3m having cell parameter a=8.365 Å. The intense peak at 30.4°, 35.7°, 43.3°, 57.5°, and 62.9° matches with the lattice plane of (220), (311), (400), (511) and (440).

FTIR data show the presence of functional groups in the iron cluster (Fig. 18d). The broad peak in the range of 3105 to 3474 cm^–1^ {as compared to the CFB nanocomposite (3311 cm^–1^), resulted from the O–H stretching caused by the hydrogen-bonded water molecules. C=C stretching bond accounted for the broad peak at around 1626 cm^–1^ whereas, the peak at 1697 cm^–1^ is the characteristic peak of the C=O bond. The bands at 1363 and 1419 cm^–1^ correspond to vibrational modes of the CH_2_ group in the iron cluster, i.e. out-of-plane (wagging) and in-plane (scissoring) bending vibrations, respectively^75^. The 1266 and 1289 cm^–1^ bands originate from the C–H bond out-of-plane bending vibrations (twisting) and O–H bond bending vibrations^75^. The peak of the Ca–Fe bond at 1028 cm^–1^ was not observed in the FTIR spectra of the iron cluster, while the emergence of a new peak at 1053 cm^–1^ corresponds to the stretching of C–S bonds^76^. The Fe–O bending caused more substantial peaks at 964 cm^–1^ in the iron cluster^77^. Based on the functional groups, probable mechanistic pathways of the formation of the iron cluster are presented in Fig. 14.

## Conclusion

Herein, a novel Ca_4_Fe_9_O_17_/Biochar (CFB) nanocomposite was fabricated via a facile green co-precipitation technique. The prepared CFB was employed for the degradation of MB and RhB dyes in single and binary pollutant system. An iron cluster was formed by the degradation products of the MB dye during the course of the reaction. The formation of iron cluster reduced the risk of secondary pollution and it could have potential biological and chemical applications like catalysis, sulfur donation, sensing, and electron transfer. The prepared CFB nanocomposite could prove to be a potential catalyst for the treatment of dye containing wastewater owing to its low cost, high availability, eco-friendlyness and high effectivity.

## Data Availability

All data generated or analyzed during this study are included in this published article.
